# 549 Length of Stay per Total Body Surface Area Burn: Validation Using the National Burn Repository

**DOI:** 10.1093/jbcr/irac012.177

**Published:** 2022-03-23

**Authors:** Kelli N Patterson, Amanda Onwuka, Rajan K Thakkar

**Affiliations:** Nationwide Children's Hospital; Ohio State University, Columbus, Ohio; Nationwide Children's Hospital, Columbus, Ohio; Nationwide Children's Hospital, Columbus, Ohio

## Abstract

**Introduction:**

A length of stay (LOS) of one day per percent total body surface area (TBSA) burn has been widely accepted in clinical practice but not validated in current pediatric burn studies. Additionally, TBSA burn data is often presented in ranges, which lacks granular details. The primary objective of this study is to validate our previous Pediatric Injury Quality Improvement Collaboration (PIQIC) findings by using a national burn registry to evaluate LOS per TBSA burn relative to burn mechanism, sociodemographic characteristics, and clinical factors which influence this ratio.

**Methods:**

We evaluated patients 0-18 years old who sustained a burn injury and whose demographics were submitted to the National Burn Repository (NBR) dataset from July 2008 through June 2018. Patients sustaining inhalation injury and electrical burns were excluded due to LOS being significantly increased unrelated to cutaneous injury. Mixed effects generalized additive regression models were then performed to identify characteristics associated with the LOS per TBSA ratio, a nonparametric variable.

**Results:**

Among 51,561 pediatric burn patients, 45% were Non-Hispanic White, 58% were male, and the median age was 3 years old (IQR: 1, 9). The most common burn mechanism was scald (55.9%), followed by flame (19.4%), and contact (16.4%), with most burns occurring at home (82.2%). The median LOS per TBSA burn ratio across all cases was 0.9 (IQR: 0.4, 1.8). In adjusted models, scald burns and radiation burns had similar LOS per TBSA burn ratios (mean predicted values: 1.22 vs 1.77), while all other burn mechanisms had a significantly higher LOS per TBSA burn ratio (p< 0.0001). Chemical burns had the highest ratio (predicted mean: 4.8), followed by contact burns (mean predicted value: 2.8; Table 1, Figure 1). Non-Hispanic White patients had lower LOS per TBSA ratios than all other races and ethnicities (p< 0.05) with mean predicted values of 1.6. Native Americans and Hispanics had the highest ratios (2.0 and 1.8 respectively, (Table 1).

**Conclusions:**

These data substantiate evidence on variance in LOS per TBSA burn relative to burn mechanism, previously demonstrated by PIQIC centers. It also validates differences in LOS per TBSA burn ratios among race/ethnicity.

